# A Multidisciplinary Algorithm for the Evaluation of Acute Neurologic Deficits Improves Management of Cryptogenic Stroke or Transient Ischemic Attack

**DOI:** 10.7759/cureus.42728

**Published:** 2023-07-31

**Authors:** Robert Hull, Nathaniel Carman, Andrew Wilson, Michael Moulton, Morgan C Jordan, Brian D Stephens, Kelvin Bush

**Affiliations:** 1 Cardiology, Brooke Army Medical Center, Fort Sam Houston, USA; 2 Neurology, Brooke Army Medical Center, Fort Sam Houston, USA

**Keywords:** algorithm, event monitor, transesophageal echocardiogram, tia, cva, stroke, cryptogenic stroke

## Abstract

The appropriate diagnosis and management of cryptogenic stroke and transient ischemic attack (TIA) is challenging and requires multidisciplinary involvement. Joint societal guidelines exist to guide the comprehensive evaluation of these entities. This study aimed to implement a standardized multidisciplinary diagnostic algorithm for cryptogenic stroke/TIA. We performed a retrospective analysis of patients admitted to the largest regional military healthcare center with stroke or TIA considered to be cryptogenic at the time of discharge. We abstracted baseline demographics and rates of extra- and intracranial imaging, transthoracic and transesophageal echocardiography, and event monitor orders at the time of discharge. The incidence of event monitor results at 30 days and six months were included. A diagnostic algorithm for evaluation of cryptogenic stroke/TIA was created and disseminated hospital-wide using increased compliance with neuroimaging, echocardiography, and cardiac rhythm monitoring as primary endpoints for our intervention. Post-intervention data abstraction revealed similar rates of extra- and intracranial imaging, but significantly greater rates of transthoracic echocardiography (70% vs. 87%, p 0.0073), inclusion of agitated saline study (41% vs. 65%, p 0.0024), and event monitors ordered at discharge (18% vs. 35%, p 0.0045). At six months there was a higher rate of event monitors obtained (24% vs. 45%, p 0.001). Our study showed implementation of an evidence-based diagnostic algorithm for evaluation of cryptogenic stroke/TIA increases appropriate use of echocardiography and event monitoring.

## Introduction

The evaluation and management of cryptogenic stroke is an evolving field with an increasing level of subspecialty multidisciplinary involvement. Depending on institutional practice these patients may be managed on a primary Neurology or Internal Medicine service. The evaluation and management of a patient with suspected cryptogenic stroke, and confirmation of the diagnosis, may involve Neurology, Neurosurgery, Cardiology, Hematology, Physical Medicine and Rehabilitation, and Internal Medicine services. Multidisciplinary care has been linked with improved outcomes and lower mortality; however, logistical challenges and appropriate utilization of resources, including appropriate consultation, is a potential problem that emerges from this paradigm [1,2].

There are joint societal Cardiology and Neurology guideline-based recommendations for the evaluation and management of stroke patients which include a Class I recommendation to establish a multidisciplinary quality improvement committee with continuous quality improvement processes [3]. Guidelines also exist regarding appropriate medical therapy for secondary prevention of stroke, as well as a Class IIa recommendation for prolonged rhythm monitoring given the high incidence of occult atrial fibrillation demonstrated in landmark studies [4-6]. Additionally, there are appropriate use criteria describing the optimal use of both transthoracic and transesophageal echocardiography in the evaluation of possible cardioembolic stroke [7].

Adding to the dynamism in this area of stroke care, several high-quality landmark trials have demonstrated patent foramen ovale (PFO) closure devices in appropriately selected cryptogenic stroke patients reduce the rate of recurrent stroke given an increased risk of paradoxical embolism in this patient subset [8,9]. We sought to evaluate the incidence of cryptogenic stroke or transient ischemic attack (TIA) at our institution to determine the appropriateness of exclusion of established stroke etiologies, and to implement a multidisciplinary outreach program with an algorithm for improving these stroke metrics.

## Materials and methods

**Figure 1:**
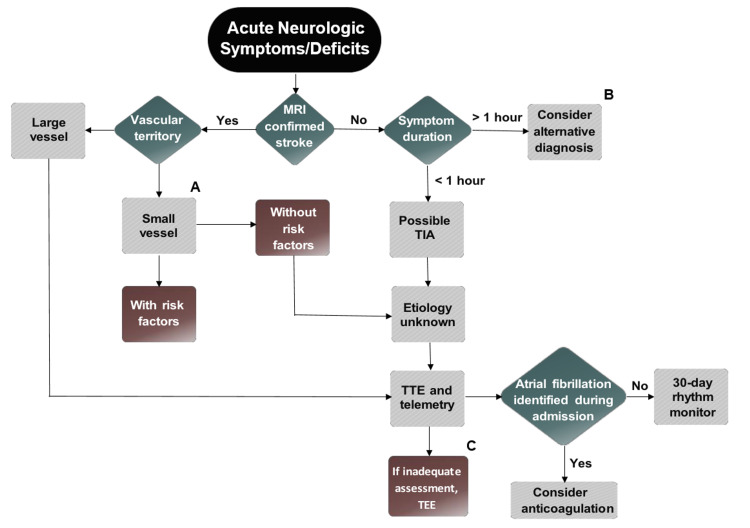
Acute Neurologic Symptoms/Deficits Algorithm A. Lacunar stroke/small vessel stroke: 1.5 cm or less (≤ 2 cm on MRI DWI) in largest dimension and in the distribution of the small penetrating cerebral arteries. B. Alternative diagnoses for transient neurologic symptoms (i.e. stroke mimics): seizure, migraine, syncope, metabolic abnormalities, electrolyte derangements, hypertensive emergency, recrudescence, medications, peripheral neuronitis/neuropathy (e.g. Bell’s palsy, vestibular neuritis, carpal tunnel), psychogenic/non-organic, etc. C. Inadequate assessment refers to either a technically limited study or a continued high index of suspicion for cardioembolic source that was not definitively ruled out by TTE. RF=risk factors (referring to tobacco, hypertension, diabetes, and hyperlipidemia); AC=anticoagulation; TTE=transthoracic echocardiogram; TEE=transesophageal echocardiogram; TIA=transient ischemic attack; MRI=magnetic resonance imaging; DWI=diffusion-weighted imaging *****There are no consensus guidelines for when to order a TTE vs a TEE, this is meant to provide a general outline but may vary on a case-by-case basis. *****All patients need telemetry monitoring and risk factor evaluation. Causes of stroke/TIA: Large artery atherosclerosis (embolus/thrombosis), cardioembolism, lacunar/small vessel occlusion, stroke of other determined etiology (e.g. dissection, vasculitis, moyamoya, etc.), stroke of undetermined etiology (cryptogenic)

We performed a retrospective review with data abstraction from a comprehensive electronic medical record of all patients admitted and discharged with stroke or transient ischemic attack from 01 August 2014 to 30 June 2018 at our tertiary care graduate medical education single-center closed referral system. The vast majority of patients were admitted to an Internal Medicine service. Non-beneficiaries seen emergently, hemorrhagic or non-cryptogenic strokes, and those who expired during the hospitalization were excluded. Baseline demographic data, relevant cardiovascular co-morbidities on presentation, and medication use at the time of discharge were collected. Review of the inpatient documentation was performed to evaluate whether a cause of stroke/TIA was determined by the time of discharge. If no etiology was determined by the time of discharge, these strokes were considered cryptogenic in nature. It was determined what imaging tests were performed including both transthoracic and transesophageal echocardiography, intracranial imaging with CT and/or MRI, and extracranial imaging with CT, MRI, and/or duplex ultrasound. In the case of echocardiography, it was noted whether this test was performed inpatient or by six months post-discharge. It was determined if event monitors were ordered by the primary team on discharge, deferred to either Cardiology or Neurology outpatient, or not included in the discharge plan. Event monitor results documented at 30 days to six months post-discharge were included. Statistical analysis was performed using IBM SPSS version 22.0 (IBM, Armonk, NY, USA). Categorical variables were compared using a Pearson’s Chi-Square test with a p-value less than 0.05 chosen to represent statistical significance. A multidisciplinary quality improvement program was developed that included a Neurology-approved algorithm for the evaluation of acute neurologic deficits with recommendations for appropriate additional testing. A multidisciplinary team of Cardiology, Internal Medicine, and Neurology performed departmental outreach reviewing cryptogenic stroke and the proposed algorithm (Figure 1) during a one-hour briefing to the Internal Medicine Residency Program. A symptom duration cutoff of one hour was used by our group in agreement with Albers et al. in the redefining of TIA using a tissue-based definition [10]. After a two-week blanking period the aforementioned data were prospectively abstracted from 18 October 2019 to 27 March 2020 with repeat data analysis performed as discussed above.

## Results

A total of 207 patients pre-intervention and 62 patients post-intervention were identified. The mean age of patients studied in the pre- and post-intervention groups was 68 and 72 years respectively. Transient ischemic attacks were more prevalent in the pre-intervention than the post-intervention group (74% pre vs. 37% post, p <0.0001). There were also significantly higher rates of hypertension and antihypertensive treatment, hyperlipidemia, diabetes, and heart failure in the post-intervention group. Full baseline characteristics are listed in Table 1.

**Table 1:** Baseline Characteristics CVA=cerebral vascular accident

Baseline Characteristics			
	Pre-Intervention (n=207)	Post-Intervention (n=62)	P-value
Male	105 (51%)	34 (55%)	0.5696
Age	68 (22-99)	72 (36-101)	0.1085
Stroke	54 (26%)	39 (63%)	<0.0001
Prior CVA	30 (14%)	12 (19%)	0.3549
Hypertension	150 (73%)	53 (85%)	0.0413
Diabetes	62 (30%)	27 (44%)	0.0459
Hyperlipidemia	113 (55%)	51 (82%)	0.0001
Heart Failure	12 (6%)	9 (15%)	0.0248
Smoking History	98 (47%)	27 (44%)	0.5992
Active Smoking	23 (11%)	6 (10%)	0.7494
Antiplatelets	188 (91%)	55 (95%)	0.274
Anticoagulation	13 (6%)	7 (11%)	0.1871
Antihypertensives	135 (65%)	50 (81%)	0.0215
Statin Therapy	182 (91%)	60 (98%)	0.0631

**Figure 2:**
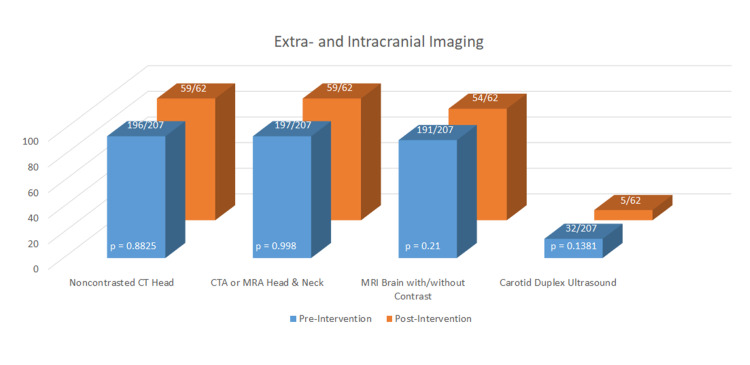
Extra- and Intracranial Imaging CTA=computed tomography angiography; MRA=magnetic resonance angiography; MRI=magnetic resonance imaging

**Figure 3:**
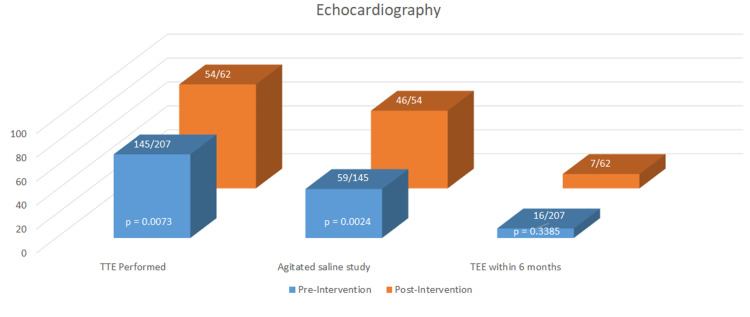
Echocardiography TTE=transthoracic echocardiography; TEE=transesophageal echocardiography

**Figure 4:**
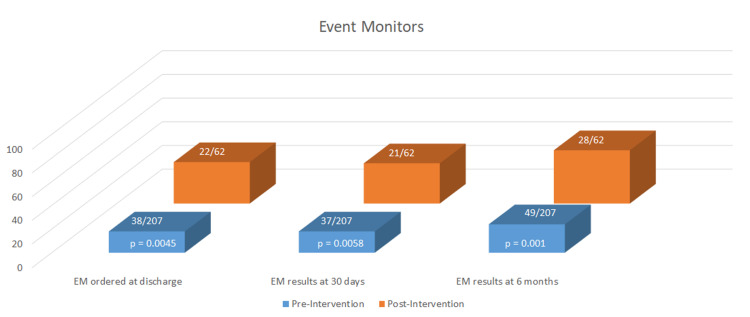
Event Monitors (EM)

The rates of extra- and intracranial imaging were high and did not significantly differ between the groups (Figure 2). Transthoracic echocardiography (TTE) was performed more in the post-intervention group (70% pre vs. 87% post, p 0.0073) and was more likely to include an agitated saline study (41% pre vs. 65% post, p 0.0024) (Figure 3). There was no difference in the incidence of transesophageal echocardiography (TEE) performed by six months and the rate of TEE in both groups was low (8% pre vs. 12% post, p 0.3385). Post-intervention, event monitors (EM) were ordered significantly more on discharge (18% pre vs. 35% post, p 0.0045), at 30 days (18% pre vs. 34% post, p 0.0058), and at six months (24% pre vs. 45% post, p 0.001) (Figure 4). If not ordered at the time of discharge, the outpatient ordering of EMs did not differ between the groups (22% pre vs. 42% post, p 0.2549). There were significantly higher rates of Neurology (62% pre vs. 76% post, p 0.043) and Cardiology (17% pre vs. 31% post, p 0.0178) consultation post-intervention.

## Discussion

Our retrospective cohort analysis demonstrated a high rate of the performance of studies to exclude intracranial disease and large vessel extracranial disease. We found low rates of performing other appropriate workup for exclusion of alternative etiologies in those discharged without a stroke etiology that persisted at six months follow-up. With a focused multidisciplinary outreach quality improvement program and an evidence-based algorithm there was substantial prospective improvement in these metrics including a 24% increase in transthoracic echocardiography, a 159% increase in agitated saline studies, and a 188% increase in event monitors obtained at six months. There was also a significant increase in subspecialist consultation and involvement.

While pre-intervention our institution achieved high rates of intracranial and extracranial imaging studies and our intervention improved upon TTE acquisition metrics, we found that we are still falling short on TEE evaluation in still undifferentiated cryptogenic stroke patients as well as event monitors despite our intervention resulting in a statistically significant improvement in this metric. These are the areas we will aim to focus improvements on in future iterations.

Our approach did not increase the rates of TEE in this cohort. This is an area to focus attention on in future iterations of our algorithm and outreach program as more research continues to demonstrate important therapeutic decisions based on results of TEE in cryptogenic stroke patients. Specifically, there is data that shows up to one in seven patients have therapy adjusted based on findings at TEE [11,12]. One major deterrent to obtaining TEE is the semi-invasive nature of the study and the perceived risks despite a well-established very low risk of major morbidity and mortality [13]. We must be cognizant however that while low risk for major complications that does not quantify the significant patient anxiety and discomfort in performing this test; a plausible reason for the apprehension in performing on some patients. The recent joint societal guidelines from the American Heart Association and American Stroke Association on secondary prevention of stroke now provide a class 2b indication for TEE in the evaluation of embolic stroke but also provide the same strength of indication for cardiac CT and cardiac MRI [14]. TEE is still widely considered the gold standard with studies demonstrating inferior sensitivity and specificity with cardiac CT for evaluation of the most common causes of cardioembolic stroke [15,16]. But given the similar strength of recommendation by current guidelines, further refinement of our algorithm should consider incorporating the use of cardiac CT or MRI as alternatives for those at high risk for complications from or contraindicated for TEE or those who desire an alternative test to increase percentage of stroke patients achieving a complete evaluation. 

We also found that it was uncommon for EMs to be ordered outpatient if not ordered at the time of discharge. We believe that the transition of care from the inpatient to the outpatient setting and the multidisciplinary involvement required in the management of these patients makes a comprehensive evaluation challenging. One barrier to this may be inpatient provider hesitancy to order these tests given concerns of ensuring timely follow-up of potentially serious test results. Our system may capture this data more accurately than an open-referral system and highlights an area of stroke care that is likely being underutilized. Based on our data we advocate for necessary studies to be ordered prior to discharge or systems established to ensure prompt outpatient completion.

An unexpected finding was a significantly lower ratio of transient ischemic attack to stroke in the post-intervention group. This may have represented improved diagnostic accuracy with our algorithm resulting in increased exclusion of non-stroke/TIA causes of acute neurologic deficits. This is supported by the increased incidence of known stroke risk factors in the post-intervention group; perhaps demonstrating less patients with transient neurologic deficits and fewer risk factors for cerebrovascular disease are being inappropriately diagnosed as TIAs. TIA can be a difficult diagnosis and this data suggests there is a wide area for improvement. While not an intended utility to our algorithm this is a use that warrants further exploration and study. We suggest more research directed at developing and validating an approach to more accurate diagnosis of TIA as increasing appropriate exclusion of patients could lead to significant decreases in cost, hospitalizations, and downstream testing.

Although prospective, these data are limited as they are non-randomized, which is a limitation of our study. As noted there are confounders that limit interpretation of this data but open possibilities for further iterations and future study.

## Conclusions

Cryptogenic stroke is a diagnosis of exclusion with joint societal guidelines on appropriate evaluation and management yet implementation of these recommendations has many barriers. In this single-center prospective, non-randomized study a multidisciplinary approach involving an outreach program with an algorithmic approach to the evaluation of cryptogenic stroke and transient ischemic attacks was implemented. This intervention resulted in improved rates of transthoracic echocardiography, agitated saline studies, and event monitors performed at six months; however, it did not increase the rates of transesophageal echocardiography. This diagnostic and management algorithm may have also improved diagnostic accuracy of TIA, and this is a further area of study to consider.
